# Stent-assisted coiling of acutely ruptured cerebral aneurysm: a multicenter prospective registry study (SAVE)

**DOI:** 10.1186/s12883-022-02800-4

**Published:** 2022-07-18

**Authors:** Gaozhi Li, Yongquan Han, Shenghao Ding, Yaohua Pan, Xiaohua Zhang, Bing Zhao

**Affiliations:** 1 Department of Neurosurgery, Renji Hospital, Shanghai Jiaotong University School of Medicine, Pujian 160 Road, Shanghai, 200217 China; 2 Department of Neurosurgery, the Affiliated Hospital of Guizhou Medical University, Guiyang, 550004 China

**Keywords:** Cerebral aneurysm, Rupture aneurysms, Stent-assisted coiling, Periprocedural complication, Prognosis

## Abstract

**Background:**

Stent-assisted coiling (SAC) has been reported as a feasible and effective treatment of wide-neck cerebral aneurysms. However, the evidence of SAC of ruptured cerebral aneurysm is lacking. There are no prospective multicenter studies regarding SAC of acutely ruptured aneurysms within 72 hours after subarachnoid hemorrhage. The purpose of the study is to evaluate the safety and efficiency of SAC of acutely ruptured cerebral aneurysms.

**Methods:**

This study is a prospective, multicenter, and observation registry of consecutive patients with acutely ruptured cerebral aneurysms treated with SAC. Acutely ruptured aneurysms were confirmed within 72 h after the onset of the syndrome. This study will enroll at least 300 patients in 7 high-volume tertiary hospitals (more than 150 cerebral aneurysms treated per year). The primary outcomes are treatment-related thromboembolic complications within 30 days of the treatment. The secondary outcomes are any hemorrhagic complications and aneurysm recurrence at 6 months of angiographic follow-up. The clinical outcomes are measured with the Modified Rankin Scale (mRS) at discharge and at the 6 months of follow-up. The favorable outcomes are defined as an mRS of grades 0 and 2.

**Discussion:**

We will perform a prospective, multicenter, and observational registry study of consecutive patients with wide-neck acutely ruptured cerebral aneurysms to improve the safety strategy of SAC of acutely ruptured cerebral aneurysms.

**Trial registration:**

Chinese Clinic Trial Registry: ChiCTR2000036972; Registration date: Aug 26, 2020

## Background

Aneurysmal subarachnoid hemorrhage (aSAH) is a fatal cerebral vascular disease, which is a worldwide health burden with high mortality and disability rates [1]. Most cerebral aneurysms rebleeding occurs in the early stage of hemorrhage and results in a high mortality and morbidity [2]. Therefore, early treatment of ruptured aneurysms has been recommended to prevent rebleeding and improve the outcome. In recent decades, endovascular treatment has been an alternative to surgical treatment of the ruptured aneurysm [3]. However, the treatment for a wide-necked ruptured aneurysm is challenging.

With the advancement of technologies, stent-assisted coiling (SAC) has been widely reported for the treatment of cerebral aneurysms, especially unruptured aneurysms with a good imaging follow-up [4, 5]. Although our previous study has shown that SAC may be safe and effective in the treatment of selected patients with acutely ruptured aneurysms [6], SAC of wide-neck ruptured aneurysms still remains controversial because of an uncertain rate of procedure-related complications and a lack of high-level clinical evidence [4, 5, 7–9].

There is no prospective multicenter data regarding the safety and efficiency of SAC for acutely ruptured aneurysms. In this study, we conducted a multicenter prospective registry of patients with acutely ruptured cerebral aneurysm treated with SAC. We will evaluate the safety and efficiency of SAC of acutely ruptured aneurysms to improve the treatment strategies and decrease the rates of treatment complications.

## Methods/design

### Study design

Stent-Assisted coiling of acutely ruptured cerebral aneurysm-multicenter prospectiVE registry (SAVE) study is a prospective, multicenter, non-randomized, and observational study of consecutive patients with acutely ruptured cerebral aneurysms treated with SAC. We included 7 high-volume tertiary hospitals (>150 cerebral aneurysms per year). Acutely ruptured aneurysms were confirmed within 72 h after the onset of the syndrome. This study was approved by the Ethics Committees of Renji Hospital, Shanghai Jiaotong University School of Medicine. Informed consents were obtained from all participants or legal representatives.

### Study aims

The purpose of the SAVE study is to evaluate the safety and efficacy of SAC of acutely ruptured aneurysms. We will investigate the rates of any treatment-related thromboembolic or hemorrhagic complications within 30 days of treatment. Besides, we will evaluate the rates of aneurysm recanalization and retreatment at 6 months of follow-up. We further will identify the risk factors of thromboembolic and hemorrhagic complications to optimize the neurointerventional regimens, including antiplatelet management (tirofiban verse traditional dual antiplatelet regimen), external ventricular drain (EVD), and patient selection.

### Participants

The study will enroll all consecutive patients with wide-necked ruptured aneurysms. All aneurysms are confirmed and treated with SAC within 72 hours of subarachnoid hemorrhage. All eligible patients were included according to the following inclusion criteria and exclusion criteria.

#### Inclusion criteria

1. Aged 18 years - less than or equal to 75 years.
2. Cerebral aneurysm confirmed by DSA, CTA, or MRA.
3. Spontaneous subarachnoid hemorrhage confirmed by a head CT scan.
4. World Federation of Neurosurgical Societies (WFNS) grade ≤ 4 grade before the treatment.
5. Ruptured wide-necked aneurysm, the neck size more than 4 mm, or the dome/ neck ratio ≤ 2.
6. Stent treatment within 0–72 hours of the onset of the syndrome.
7. Ruptured aneurysms treated with SAC.
8. Informed consents were obtained from patients or legal representatives.

#### Exclusion criteria

1. Patients with a modified Rankin Score (mRS) of grade more than 3 prior to the hemorrhage.
2. Intracerebral hematoma greater than 20 ml shown on CT scan
3. Pregnant or lactating women.
4. Patients with other systemic refractory diseases such as tumor and hepatopathy.
5. Patients with other serious systemic diseases, acute myocardial infarction, renal dysfunction, malignant tumor.

### Detailed clinical data

We prospectively collect clinical variables, including geographic information, medical records, clinical condition of World Federation of Neurosurgical Societies (WFNS) grade, imaging finding of Fisher grade, aneurysm characteristics, procedural reports, immediate angiographic results, any treatment-related complications, radiological dataset and clinical outcomes at discharge and 6 months of follow-up. These variables are collected by the independent neurosurgeons who are not involved in the procedure.

### Data management

Data are recorded in an electronic case report form (eCRF). The additional relevant documentations are deposited in digital images and communication in medicine format and medical notes. Handwriting files (consent forms, case report files from the participants) are kept secured at appropriate sites. All data pertaining to this study will be available to quality assurance board, ethics board and other regulatory entities.

### SAC treatment protocol

Endovascular treatment is preferred in patients with anterior circulation aneurysms which are suitable for both endovascular coiling or surgical clipping and cases with posterior circulation aneurysms. SAC is considered for wide-neck aneurysms (neck size ≥4 mm or dome to neck ratio ≤ 2) if the single coiling influences the parent artery or fails the embolization when using the dual microcatheters or other techniques. The balloon-assisted coiling is not routinely used in all the centers.

The stent type is chosen dependent on the institutional standards of practice. Stents are recommended to be placed through semi-jailing in which the stent is partially deployed to cover the aneurysm neck. EVD is considered in patients with acute hydrocephalus or severe intraventricular hemorrhage after the successful coiling. All patients are treated under general anesthesia and were transferred to the neurosurgical intensive care unit (NICU) and treated with standard management for aneurysmal subarachnoid hemorrhage [3].

### Antiplatelet management

A bolus of 50–75 IU/kg of heparin is used for general heparinization. Intermittent boluses of 1000 IU are given per hour during the procedure. Activated clotting time is recommended to maintain at two-three times of the baseline. There are two standard protocols for antiplatelet therapy for the stent deployment One is a loading dose of 300 mg clopidogrel and 300 mg of aspirin given through a nasogastric tube or rectally before 2 hours of the stent usage. The other is an 8–10 u g/kg loading dose of tirofiban for 3 min administered intravenously during the stent deployment followed by a 0.10 μg/kg/min maintenance [6, 10, 11]. A dose of 100 mg aspirin and 75 mg clopidogrel (traditional dual antiplatelet therapy) is given daily for 3 months after the procedure. The platelet function testing is recommended to be measured after the dual antiplatelet therapy.

### Outcome measurement and follow-up

The primary outcome is any thromboembolic complications within 30 days after the treatment. Thromboembolic complications are defined as parent artery or distal artery luminal filling defects, or vascular territory infarction confirmed by CT scan or MRI scan which were not presented on previous radiological examination [12]. Vasospasm related ischemia is excluded from the complication.

The secondary outcomes are hemorrhagic complications and aneurysm recurrence. The hemorrhagic complications are defined as any angiographic contrast extravasation and new hemorrhage or aneurysm rebleeding [13]. Angiographic outcomes are determined by the Raymond grade on immediate and follow-up angiographies. The aneurysm complete occlusion is defined as a Raymond grade of I. Aneurysm recurrence measured is defined as any angiographic deterioration in the Raymond grade from initial grade after the treatment to the 6 months follow-up.

**Fig. 1:**
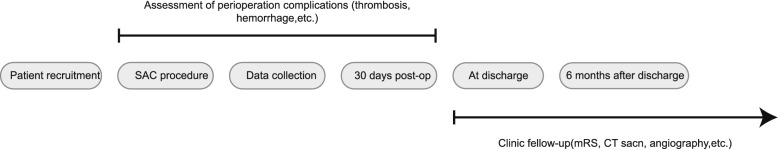
Patient flow diagram

Clinical outcomes are measured with the mRS score at discharge and 6 months after the treatment. The favorable outcomes are defined as an mRS of grades 0 and 2. The clinical outcomes are measured by an independent neurosurgeons using the telephone interview, in-person interview, or hospitalization follow-up. An overall of work flow is depicted in Fig. 1.

### Sample size

According to previous literatures, the rate of periprocedural complications after SAC for ruptured aneurysms is assumed as 12% [14]. The number of patients enrolled in the registry was estimated to be at least 300 with a two-sides significance level of 5% and a power of 80%. An expected number of patients was 315 because 5% of patients is expected to be loss of follow-up.

### Statistical analysis

Statistical analysis will be performed with IBM SPSS version 25.0 (IBM SPSS; Armonk, NY, US). Continuous variables are presented as mean ± standard deviation and categorical variables are presented as frequency or percentage. The Chi-square test and Fisher’s precision test are used to compare categorical variables. T-test and Wilcoxon’s rank-sum test are used for continuous variables. The univariate and multivariate logistic regression analyses are used to identify the risk factors of perioperative complications. 95% confidence interval (CI) and the odds ratio (OR) are calculated. The statistical significance is considered when a *P* value is < 0.05.

### Study status

The SAVE study is in the recruitment stage. The first case was enrolled in the registry on Sept 26, 2020.

## Discussion

Endovascular treatment has been an important treatment modality for ruptured aneurysms. International Subarachnoid Aneurysm Trial (ISAT) has shown that endovascular coiling of ruptured aneurysm had lower mortality and disability at 1 year compared with microsurgical clipping (23.5% vs 30.9%) [15]. The Barrow Ruptured Aneurysm Trial has shown that endovascular coiling resulted in fewer unfavorable outcomes when comparing the safety of clipping and coiling for ruptured aneurysms [16]. However, the endovascular coiling of ruptured wide-necked aneurysms remains challenging because of the probability of the coil protruding into the parent artery causing serious complications.

SAC alters hemodynamics characters of parent artery and aneurysms and contributes to thrombosis in the aneurysms to decrease the risk of recurrence of aneurysms [5, 7]. Several previous studies have shown the safety and efficacy of SAC for ruptured aneurysms [5, 7, 17, 18]. However, most studies involved a wide range of time intervals after the onset of hemorrhage. Although SAC of acutely ruptured cerebral aneurysms is feasible, the sizes of samples were relatively small in retrospective studies [4, 19–21]. A single-center retrospective study that reported the safety and efficacy of SAC of ruptured cerebral aneurysms (no more than 28 days of hemorrhage) has shown that the rates of periprocedural complications and mortality in SAC group did not differ from those of non-SAC group (overall perioperative complications: 8.3% vs 4.5%, *P* = 0.120; mortality:1.5% vs 0.7%, *P* = 0.796), which accompanied with a higher occlusion rate and lower recurrence rate in SAC group (82.5% vs 66.7, 3.5% vs 14.5%, *p* = 0.007) [22]. Meanwhile, Xue et al [23] conducted a retrospective study on the safety and efficacy of LVIS-assisted coiling of acutely ruptured wide-necked intracranial aneurysms (no more than 28 days) compared with a single coiling strategy, which showed a similar result of perioperative complications of 7.6%. A current systematic review enrolling 399 patients who received SAC of a ruptured cerebral aneurysm (less than 7 days after onset) reported approximately 12% of overall perioperative complications [14]. Nevertheless, a meta-analysis including 499 patients with ruptured cerebral aneurysms who underwent SAC has shown that the rate of adverse events was approximately 20.2% [24].

The evidence of SAC of acutely ruptured cerebral aneurysm is lacking. Meanwhile, there is no prospective multicenter study regarding the genuinely acutely ruptured aneurysms defined as within 72 hours after the onset of the syndrome. The safety and efficiency of SAC of acutely ruptured cerebral aneurysms require further prospective study. Many detailed questions including the optimal time for early treatment, the appropriate regimen of antiplatelet therapy, and risk factors for perioperative complications remain unknown [25–27]. In this study, we will perform a prospective, multicenter, and observational registry study of consecutive patients with wide-neck acutely ruptured cerebral aneurysms to improve the safety strategy of SAC of acutely ruptured cerebral aneurysms.

## Data Availability

The datasets used and/or analyzed during the current study are available from the corresponding author on reasonable request.

## Abbreviations

SAC: Stent-assisted coiling
aSAH: Aneurysmal subarachnoid hemorrhage
SAVE: Stent-Assisted coiling of acutely ruptured cerebral aneurysm-multicenter prospectiVE registry study
mRS: Modified Rankin Scale
EVD: External ventricular drain
DSA: Digital subtraction angiography
CTA: Tomography angiography
MRA: Magnetic resonance angiography
WFNS: World Federation of Neurosurgical Societies
eCRF: Electronic case report form
NICU: Neurological intensive care unit
ISAT: International Subarachnoid Aneurysm Trial
